# Commentary: The framework for systematic reviews on psychological risk factors for persistent somatic symptoms and related syndromes and disorders (PSY-PSS)

**DOI:** 10.3389/fpsyt.2023.1270497

**Published:** 2023-08-28

**Authors:** Sabrina Berens, Johannes C. Ehrenthal, Jonas Tesarz

**Affiliations:** ^1^Independent Researcher, Heidelberg, Germany; ^2^Department of Psychology, University of Cologne, Cologne, Germany; ^3^Department of General Internal Medicine and Psychosomatics, University Hospital Heidelberg, Heidelberg University, Heidelberg, Germany

**Keywords:** persistent somatic symptoms, somatic symptom disorder, psychological risk factors, irritable bowel syndrome, psychodynamic, mentalizing, psychic structure, personality functioning

## Introduction

A framework (1) for research on psychological risk factors for persistent somatic symptoms (PSS) is a milestone in stimulating and improving future research in this area, and we appreciate the attempt to systematize the complex field of PSS by defining relevant patient groups and psychological variables. In their work, the authors attempt to compile a comprehensive list of search terms for further systematic reviews on psychological risk factors for PSS and PSS-related outcomes (PSY-PSS). However, the paper omits factors related to personality functioning and mentalizing (2, 3) that are highly relevant for the understanding and treatment of patients with PSS (4–8). In this commentary, we therefore want to outline further areas of psychological risk factors that have already shown their significance for patients with somatic symptom disorder (SSD) and in other PSS-related syndromes and disorders, such as irritable bowel syndrome (IBS).

## Risk factors in patients with PSS related to personality and personality functioning

First, we would like to acknowledge that a number of factors related to personality are already integrated in the list provided by the authors (1). Notably, the reception of alexithymia, emotion regulation, adverse childhood events, parent-child relationship, and interpersonal factors does also partly include a perspective of PSS as a disorder related to attachment and affective processing (9). The concept of alexithymia especially refers to an emotional blindness with specific deficits in distinguishing and expressing emotional states (10). Relatedly, the cognitive-developmental model of emotional awareness conceptualizes different states of emotional understanding (11) and has been evaluated in patients with somatoform disorders (12). Alexithymic characteristics in this patient group describe the difficulty of identifying and describing feelings that go along with the misinterpretation of bodily sensations that accompany emotional arousal (13). In addition to individual deficits, emotional avoidance often fulfills an interpersonal function in patients with PSS (14). However, modern approaches describe phenomena of alexithymia regarding affective processing as deficits in embodied mentalization (15), deficits in affective theory of mind (16), or as specific deficits of basic abilities such as affect differentiation or affect tolerance (3), all of which share an explicit or implicit dimensional model of personality functioning. Surprisingly, these aspects are missing in the presented framework (1).

## Empiric evidence of deficits in mentalization and personality functioning

In the next section, we will briefly outline the importance of personality-oriented variables such as mentalizing or—more broadly speaking—personality functioning for understanding and treating patients with PSS. According to the concept of embodied mentalization, a main problem of patients with PSS is to distinguish emotional and physical states from each other and to classify them in a reflective way (15). Mentalization capacity is shaped by early mirroring and containing processes within the parent-child-relationship (2). A prospective study showed a significant negative relationship between maternal sensitivity at 18 months and somatization at age 5 years as well as a strong connection between attachment anxiety and health anxiety in adulthood (17). From this perspective, current psychodynamic therapies for patients with PSS have been derived, which aim at three key systems: attachment, mentalizing, and impairments in epistemic trust (18). This adds to other meta-analytic evidence for the effectiveness of psychodynamic approaches in functional somatic disorders (19). Furthermore, a recent systematic review stresses the need to integrate dimensional assessment of personality functioning in patients with SSD (20). The concept of structural integration or personality functioning has many similarities with the DSM-5 AMPD and ICD-11 approach for personality disorders (21). Empirically, personality functioning served as a mediator between childhood maltreatment and somatic symptoms and mental health due to capacities of self-reflection, regulation and identity formation (22). Finally, personality functioning can be directly used for tailored treatment planning (23, 24) and therefore should not be neglected in research about PSS.

More specifically, the importance of deficits in mentalizing and personality functioning, especially specific structural deficits in affective processing, are empirically well-supported for patients with PSS: In recent studies, patients with IBS showed higher mentalizing deficits than healthy controls (4, 8) and patients with inflammatory bowel diseases (4). Additionally, in gastroenterological patients, the diagnostic B-criterion of SSD was associated with higher mentalizing deficits and deficits in personality functioning according to the Operationalized Psychodynamic Diagnosis System (6). Furthermore, experimental research indicates that higher order emotional awareness, which includes comprehension as a form of mentalization compared to symptom attention without comprehension, protects from somatic complaints (7). A further study compared patients with IBS and healthy controls according to specific dimensions of affective processing and showed that the deficits are especially prominent in understanding and tolerating difficult affective states captured as affect tolerance and affect differentiation (5). Both are subscales of the OPD-SQ (25), which is recommended as a measurement tool in a narrative systematic review on emotion regulation processes in SSD and related conditions (26). From these results, it was hypothesized that deficits in understanding and regulating physical and emotional states—as it is part of the mentalization and personality functioning concept—are functionally connected to increased anxiety, worry, and behavioral preoccupations in patients with SSD (6).

## Discussion

In this *General Commentary* we report the empiric evidence of deficits in mentalization and specific structural deficits in affective processing in patients with PSS and related syndromes as IBS. So far, mentalization, reflective functioning, embodied mentalization, affective theory of mind, psychic structure, personality functioning, affect tolerance and affect differentiation are not integrated as search terms in the referred systematic review (1). Empirical evidence tentatively suggests their relevance for patients with PSS, including related empirically supported treatment models, which would otherwise be excluded, and therefore we argue for their inclusion. Overall, we appreciate the effort put forth by Hüsing et al. and believe that by including these additional psychological risk factors, their framework could serve as an even more valuable resource for researchers, clinicians, and other stakeholders in the field of PSS.

## Author contributions

SB: Writing—original draft. JE: Writing—review and editing. JT: Writing—review and editing.

## References

[1] Hüsing P Smakowski A Löwe B Kleinstäuber M Toussaint A Shedden-Mora MC. The framework for systematic reviews on psychological risk factors for persistent somatic symptoms and related syndromes and disorders (PSY-PSS). Front Psychiatry. (2023) 14:1142484. 10.3389/fpsyt.2023.1142484 37091694 PMC10113674

[2] Fonagy P Gergely G Jurist EL Target M. Affect Regulation, Mentalization and the Development of the Self . New York, NY: Routledge (2018).16602354

[3] Rudolf G. Strukturbezogene Psychotherapie: Leitfaden zur psychodynamischen Therapie struktureller Störungen. Stuttgart: Schattauer Verlag (2020).

[4] Berens S Schaefert R Baumeister D Gauss A Eich W Tesarz J. Does symptom activity explain psychological differences in patients with irritable bowel syndrome and inflammatory bowel disease? Results from a multi-center cross-sectional study. J. Psychosomat. Res. (2019) 126:109836. 10.1016/j.jpsychores.2019.109836 31627144

[5] Berens S Schaefert R Ehrenthal JC Baumeister D Eich W Tesarz J. Different dimensions of affective processing in patients with irritable bowel syndrome: a multi-center cross-sectional study. Front Psychol. (2021) 12:926. 10.3389/fpsyg.2021.625381 33854462 PMC8039143

[6] Berens S Schaefert R Ehrenthal JC Baumeister D Gauss A Eich W. The validity of somatic symptom disorder in patients with gastrointestinal complaints. J Clin Gastroenterol. (2021) 55:e66–76. 10.1097/MCG.0000000000001505 33780221

[7] Ballespí S Vives J Alonso N Sharp C Ramírez MS Fonagy P. To know or not to know? Mentalization as protection from somatic complaints. PLoS ONE. (2019) 14:e0215308. 10.1371/journal.pone.0215308 31048857 PMC6497236

[8] Dzirlo L Richter F Steinmair D Löffler-Stastka H. Reflective functioning in patients with irritable bowel syndrome, non-affective psychosis and affective disorders—Differences and similarities. Int J Environ Res Public Health. (2021) 18:2780. 10.3390/ijerph18052780 33803368 PMC7967285

[9] Waller E Scheidt CE. Somatoform disorders as disorders of affect regulation: a development perspective. Int Rev Psychiatry. (2006) 18:13–24. 10.1080/09540260500466774 16451876

[10] Sifneos PE. The prevalence of ‘alexithymic'characteristics in psychosomatic patients. Psychother Psychosom. (1973) 22:255–62. 10.1159/000286529 4770536

[11] Lane RD Quinlan DM Schwartz GE Walker PA Zeitlin SB. The Levels of Emotional Awareness Scale: a cognitive-developmental measure of emotion. J Pers Assess. (1990) 55:124–34. 10.1080/00223891.1990.9674052 2231235

[12] Subic-Wrana C Beutel ME Knebel A Lane RD. Theory of mind and emotional awareness deficits in patients with somatoform disorders. Psychosom Med. (2010) 72:404–11. 10.1097/PSY.0b013e3181d35e83 20223925

[13] Taylor GJ Bagby RM Parker JD. Disorders of Affect Regulation: Alexithymia in Medical and Psychiatric Illness. London: Cambridge University Press (1999).

[14] Krivzov J Baert F Meganck R Cornelis S. Interpersonal dynamics and therapeutic relationship in patients with functional somatic syndromes: a metasynthesis of case studies. J Counsel Psychol. (2020) 68:593–607. 10.1037/cou0000529 32852968

[15] Luyten P Van Houdenhove B Lemma A Target M Fonagy P. A mentalization-based approach to the understanding and treatment of functional somatic disorders. Psychoanal Psychother. (2012) 26:121–40. 10.1080/02668734.2012.678061

[16] Stonnington CM Locke DE Hsu C-H Ritenbaugh C Lane RD. Somatization is associated with deficits in affective Theory of Mind. J Psychosom Res. (2013) 74:479–85. 10.1016/j.jpsychores.2013.04.004 23731744

[17] Maunder RG Hunter JJ Atkinson L Steiner M Wazana A Fleming AS. An attachment-based model of the relationship between childhood adversity and somatization in children and adults. Psychosom Med. (2017) 79:506–13. 10.1097/PSY.0000000000000437 27941580

[18] Luyten P Fonagy P. Psychodynamic psychotherapy for patients with functional somatic disorders and the road to recovery. Am J Psychother. (2020) 73:125–30. 10.1176/appi.psychotherapy.20200010 33203227

[19] Abbass A Town J Holmes H Luyten P Cooper A Russell L. Short-term psychodynamic psychotherapy for functional somatic disorders: a meta-analysis of randomized controlled trials. Psychother Psychosom. (2020) 89:363–70. 10.1159/000507738 32428905

[20] Macina C Bendel R Walter M Wrege JS. Somatization and somatic symptom disorder and its overlap with dimensionally measured personality pathology: a systematic review. J Psychosom Res. (2021) 151:110646. 10.1016/j.jpsychores.2021.110646 34715494

[21] Zimmermann J Ehrenthal JC Cierpka M Schauenburg H Doering S Benecke C. Assessing the level of structural integration using operationalized psychodynamic diagnosis (OPD): implications for DSM−5. J Pers Assess. (2012) 94:522–32. 10.1080/00223891.2012.700664 22808938

[22] Krakau L Tibubos AN Beutel ME Ehrenthal JC Gieler U Braehler E. Personality functioning as a mediator of adult mental health following child maltreatment. J Affect Disord. (2021) 291:126–34. 10.1016/j.jad.2021.05.006 34034217

[23] Ehrenthal JC Benecke C. “Tailored treatment planning for individuals with personality disorders: the operationalized psychodynamic diagnosis (OPD) approach,” In:Kramer U, Editor. Case Formulation for Personality Disorders. Cambridge, MA: Elsevier (2019). p. 291–314.

[24] Bach B Tracy M. Clinical utility of the alternative model of personality disorders: a 10th year anniversary review. Person Disord Theory Res Treat. (2022) 13:369. 10.1037/per0000527 35787123

[25] Ehrenthal JC Dinger U Horsch L Komo-Lang M Klinkerfuß M Grande T. Der OPD-Strukturfragebogen (OPD-SF): Erste Ergebnisse zu Reliabilität und Validität. Psychother Psychosom Med Psychol. (2012) 62:25–32. 10.1055/s-0031-1295481 22271173

[26] Schnabel K Petzke TM Witthöft M. The emotion regulation process in somatic symptom disorders and related conditions-A systematic narrative review. Clin Psychol Rev. (2022) 97:102196. 10.1016/j.cpr.2022.102196 36063620

